# Design of Wood-Based Gd (III)-Hemoporphyrin Monomethyl Ether Eco-Material for Optical Oxygen Sensing with a Wide Detection Range

**DOI:** 10.3390/s25061670

**Published:** 2025-03-08

**Authors:** Yujie Niu, Jinxin Wang, Zhongxing Zhang, Ting Liu

**Affiliations:** Department of Physics, Northeast Forestry University, Harbin 150040, China; niuyujie@nefu.edu.cn (Y.N.); wangjinxin@nefu.edu.cn (J.W.); cqxnh@nefu.edu.cn (Z.Z.)

**Keywords:** balsa wood, gadolinium, porphyrin, oxygen-sensing, optical parameter

## Abstract

Gaseous oxygen detection is essential in numerous production and manufacturing sectors. To meet the varying oxygen detection requirements across different fields, techniques that offer a wide oxygen detection range should be developed. In this study, a wood-based oxygen sensing material was designed using balsa wood as the supporting matrix and gadolinium hemoporphyrin monomethyl ether (Gd-HMME) as the oxygen-sensitive indicator. The wood-based Gd-HMME exhibits a cellular porous structure, which not only facilitates the loading of a substantial number of indicator molecules but also enables the rapid interaction between indicators and oxygen molecules. *OP* is defined as the ratio of the phosphorescence intensity of the oxygen-sensing material in the anaerobic and aerobic environment. A linear relationship between *OP* and oxygen partial pressure ([*O*_2_]) was obtained within the whole range of [*O*_2_] (0–100 kPa). The wood-based Gd-HMME exhibited excellent resistance to photobleaching, along with a rapid response time (3.9 s) and recovery time (4.4 s). It was demonstrated that the measurement results obtained using wood-based Gd-HMME were not influenced by other gaseous components present in the air. An automatic oxygen detection system was developed using LabVIEW for practical use, and the limit of detection was determined to be 0.01 kPa.

## 1. Introduction

Gaseous oxygen plays a critical role in numerous key sectors, including chemistry, the food industry, manufacturing, agriculture, and construction, etc. [1,2,3]. Precise gaseous oxygen detection is essential in the relevant fields, for example, enhancing crop yields and the quality of industrial products, as well as preventing potential risks in industrial production processes [4,5]. The detection range of oxygen concentration varies significantly across diverse application fields [6,7]. Therefore, continuous and real-time monitoring of oxygen concentration across a wide range is crucial to satisfy the varied demands of different fields [8]. Traditional methodologies employed in oxygen detection include the absorption spectroscopy method, Winkler titration method, and the Clark electrode method [9,10,11]. Due to the relatively small absorption cross-section of oxygen molecules, the signal-to-noise (SNR) ratio in oxygen concentration detection using the absorption spectroscopy method is inherently low. Consequently, highly sensitive detection equipment is necessary, which increases the economic investment for this method [9,12,13]. Besides, oxygen is consumed during the detection process using the Winkler titration method and Clark electrode method, which has a significant effect on the outcomes [10,11], and environmental factors also exert a considerable influence on the results [14,15]. To address the limitations of these methods, optical oxygen sensors based on the principle of oxygen-induced fluorescence quenching have been developed [16,17,18]. Compared with traditional oxygen detection methods, optical oxygen sensors exhibit significant advantages, such as superior signal-to-noise ratio [19], enhanced sensitivity [20], rapid response time [21], robust anti-interference performance, and broad applicability [22]. Consequently, they have garnered considerable attention from both academia and industry.

Optical oxygen sensors generally comprise an oxygen-sensitive indicator molecule and a substrate material that is used to support the indicators [23,24]. Polypyridyl transition metal complexes and metalloporphyrins are usually utilized as indicator molecules in optical oxygen sensors due to their significant stokes shift and remarkable sensitivity to oxygen [24,25]. The indicator molecules are encapsulated within porous matrix materials, such as sol–gel, polymer, filter paper, etc. [26,27,28]. The optical oxygen sensors are subsequently developed. Most oxygen sensors can effectively operate within a defined range of oxygen concentrations, which is determined by the selection of specific indicator and substrate materials. However, the limited detection range constrains the overall applicability of these sensors. Therefore, there is a need to develop optical oxygen sensors that offer a wider detection range. 

To achieve this objective, oxygen-sensitive molecules that exhibit high luminescence quantum yields and substantial stokes shifts should be chosen as the indicator molecules, and materials with a large-porous structure and stable chemical properties should be chosen as the supporting matrix. In recent studies, gadolinium-labeled hematoporphyrin monomethyl ether (Gd-HMME) was developed and demonstrated to have high phosphorescent quantum yields due to the heavy atom effect [29,30]. It was demonstrated that Gd-HMME exhibited excellent oxygen sensitivity on filter paper and in methanol solution, making it a promising candidate as an oxygen-sensitive indicator molecule [28,31,32]. 

Although a variety of materials have been developed as a supporting matrix for oxygen sensors, environmentally friendly and efficient materials are still being pursued to be used as the supporting matrix. Natural wood possesses extensive and large porous structures, stable chemical and mechanical properties, as well as ecological environmental benefits, making it an excellent choice for use as the supporting matrix for optical oxygen sensors [33,34]. However, research exploring the use of wood as the supporting material for optical oxygen sensors is nonexistent.

Among the various types of wood, balsa wood exhibits thicker and more porous internal conduits due to its specific growing environment [35]. In addition, it exhibits a lower density and weight, while maintaining relatively high strength, stiffness, and compressive resistance [36]. These advantages make balsa wood an excellent supporting matrix for optical oxygen sensors. In this study, porous balsa wood has been investigated as a potential supporting matrix for an optical oxygen sensor, using Gd-HMME as the oxygen indicator molecule, with the goal of preparing a wood-based Gd-HMME oxygen sensor. *OP* was defined as the ratio of phosphorescence intensity in anaerobic conditions to that in aerobic conditions. The dependence of *OP* on oxygen partial pressure ([*O*_2_]) using wood-based Gd-HMME was determined. The impact of photobleaching behavior, response time, component gases in the air, and the relative humidity (RH) on the sensor’s detection performance were systematically investigated. Meanwhile, the long-term stability of the material was evaluated. A software program developed using LabVIEW (version 2018) was designed to measure the real-time oxygen concentration using the wood-based Gd-HMME material. The fluctuations and detection limits of the automatic oxygen detection system were also assessed. Finally, the system was applied to real samples to evaluate its accuracy.

## 2. Experimental Section

### 2.1. Materials

Hematoporphyrin monomethyl ether (HMME) was purchased from Shanghai Xianhui Pharmaceutical Co., Ltd., (Shanghai, China). Anhydrous gadolinium chloride (GdCl_3_) and imidazole were obtained from Shanghai Aladdin Biochemical Technology Co., Ltd., (Shanghai, China). Methanol was purchased from Tianjin Fuyu Fine Chemical Co., Ltd., (Tianjin, China). High-purity nitrogen and oxygen were purchased from Harbin Tongda Co., Ltd., (Harbin, China). Balsa wood was obtained from Harbin Haicheng Wood Processing Plant (Harbin, China).

### 2.2. Preparation of Wood-Based Gd-HMME Oxygen Sensing Material

Gd-HMME was synthesized through the subsequent procedures [32]: GdCl_3_ (50 mg), HMME (12 mg) and an excess of imidazole (6 g) were simultaneously introduced into a 100 mL three-necked flask. The reactants were placed on a magnetic stirrer set at 300 revolutions per minute for 2.5 h. The aforementioned reaction process was conducted under a nitrogen-protected atmosphere. The product was then cooled to room temperature and subsequently dissolved in a methanol solution, resulting in the formation of a Gd-HMME solution. Flaky-like balsa wood with a dimension of 10 × 10 × 0.8 mm^3^ (longitudinal × tangential × radial) was impregnated with the Gd-HMME solution and then dried at room temperature for 24 h to develop the wood-based Gd-HMME material. All procedures were conducted under dark conditions.

### 2.3. Instruments and Characterization

The UV–vis absorption spectra of HMME and Gd-HMME were measured using a deuterium lamp as the light source. The methanol solutions of HMME and Gd-HMME were placed in cuvettes with a length of 1 cm for measurements. A miniature fiber optic spectrometer (QE65000, Ocean Optics, Orlando, FL, USA) was used to record the signal. The photoluminescence spectra of all the samples were measured using a 405 nm diode laser as the excitation source, and the signal was recorded by a miniature fiber optic spectrometer (USB2000, Ocean Optics, Orlando, FL, USA). The morphological characteristics of unimpregnated balsa wood and wood-based Gd-HMME were investigated using scanning electron microscopy (SEM, JSM–7500F, JEOL Ltd., Akishima, Japan). Elemental analysis and mapping of wood-based Gd-HMME were performed using an energy dispersive spectrometer (EDS, Oxford X–Max 20, Oxford Instruments, Abingdon, Oxfordshire, UK) attached to the SEM.

High-purity nitrogen and oxygen are introduced into a gas mass flow controller (Sevenstar D07-19B, BeiJing Sevenstar Flow Co., Ltd., Beijing, China) to achieve oxygen with different partial pressures. The gas mixture was then directed into an air chamber equipped with an air inlet on one end and a gas check valve on the opposite end. The samples were positioned within the optical window of the air chamber, and the phosphorescence intensity was measured in different [*O*_2_] under the irradiation of a 405 nm laser. This process allows for the determination of the relationship between *OP* and [*O*_2_]. 

Balsa wood was immersed in methanol solutions of Gd-HMME at varying concentrations to investigate the impact of the Gd-HMME concentration on the relationship between *OP* and oxygen levels. The photobleaching behavior was evaluated by continuously illuminating the sample for 3600 s using a 405 nm laser at a power density of 1.5 mW/cm^2^ in a high-purity nitrogen atmosphere. Response time and recoverability were assessed by monitoring the changes in phosphorescence intensity of the sample during transitions between air and pure nitrogen environments. 

To examine the influence of air humidity on the performance of the wood-based Gd-HMME, the dependence of *OP* on [*O*_2_] using the wood-based Gd-HMME material was evaluated under relative humidity conditions of 15%, 35%, 55%, 75%, and 95%, respectively. The various relative humidity conditions were achieved by introducing water vapor into the chamber. Methane (CH_4_), hydrogen (H_2_), argon (Ar), carbon dioxide (CO_2_), and carbon monoxide (CO) were mixed with oxygen to form different gas mixtures. The relationships between *OP* and [*O*_2_] in these different gas mixtures were investigated to evaluate the specificity of the wood-based Gd-HMME material used for oxygen detection. In addition, the wood-based Gd-HMME material was stored in the dark for 7 days, and the relationship between *OP* and [*O*_2_] were obtained for the fresh preparation, as well as after 1, 3, and 7 days of storage to evaluate the long-term stability of the material.

Signal fluctuations using the wood-based Gd-HMME material for monitoring oxygen levels was evaluated. The *OP* values at each [*O*_2_] were recorded 20 times, based on the calibration curve derived from the preceding experiment, and fluctuations at different oxygen concentrations were analyzed to determine the measurement uncertainty for each [*O*_2_]. The uncertainty was defined as half the difference between the maximum and minimum measured values. Air samples were collected at specific time points throughout the day (7:00, 11:00, 15:00, 19:00, and 23:00) to evaluate the accuracy of the automatic detection system.

## 3. Results and Discussion

### 3.1. Optical Properties of Gd-HMME in Methanol Solution

**Figure 1:**
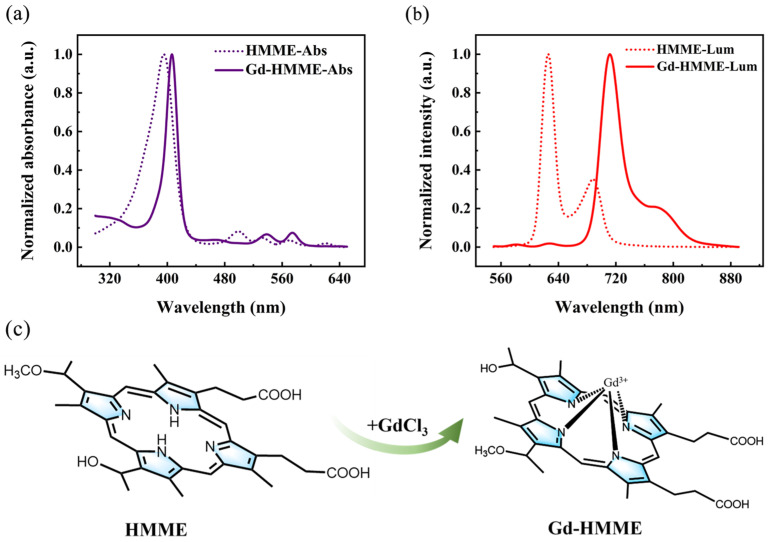
(**a**) UV–visible absorption spectra. (**b**) Photoluminescence spectra of HMME and Gd-HMME in methanol solution. (**c**) Chemical structures of HMME and Gd-HMME.

The optical properties of Gd-HMME were investigated through the measurement of UV–vis absorption and photoluminescence spectra, and HMME was measured for comparison. The absorption spectrum of HMME (dashed line) and Gd-HMME (solid line) are shown in Figure 1a. The absorption spectrum of HMME exhibits a Soret band within the range of 300 nm to 447 nm, along with four distinct Q-bands centered at 500 nm, 532 nm, 570 nm, and 620 nm, respectively. In the absorption spectrum of Gd-HMME, the Soret band spans from 358 nm to 440 nm. In comparison with that of HMME, the Soret band of Gd-HMME exhibits a narrower bandwidth and is accompanied by a redshift of approximately 11 nm, primarily due to the out-of-plane molecular structure of Gd-HMME [30]. In addition, the Q-bands were reduced to two in a range of 514 nm to 558 nm and 558 nm to 591 nm, which was attributed to the increased molecular symmetry from D_2h_ (HMME) to C_4v_ (Gd-HMME) resulting from Gd^3+^ ion chelation [37]. The photoluminescence spectra of HMME (dashed line) and Gd-HMME (solid line) are presented in Figure 1b. HMME exhibits two fluorescence emission peaks centered at 625 nm and 689 nm, which have been extensively reported in the literature [38,39]. In contrast, the photoluminescence spectrum of Gd-HMME features four emission peaks. Among these, two weak fluorescence peaks are observed at 582 nm and 627 nm, while two intense phosphorescence peaks appear at 711 nm and 790 nm. The phosphorescence emissions are attributed to the heavy atom effect induced by the Gd^3+^ ion [29]. The chemical structures of HMME and Gd-HMME are presented in Figure 1c. The changes in the absorption and photoluminescence spectra of HMME and Gd-HMME indicate that the HMME molecules have fully combined with Gd^3+^ ions. The absence of characteristic HMME luminescence in the Gd-HMME spectra suggests no residual HMME in the samples, thereby confirming the successful synthesis of high-purity Gd-HMME.

### 3.2. Optical Properties of Wood-Based Gd-HMME Material

**Figure 2:**
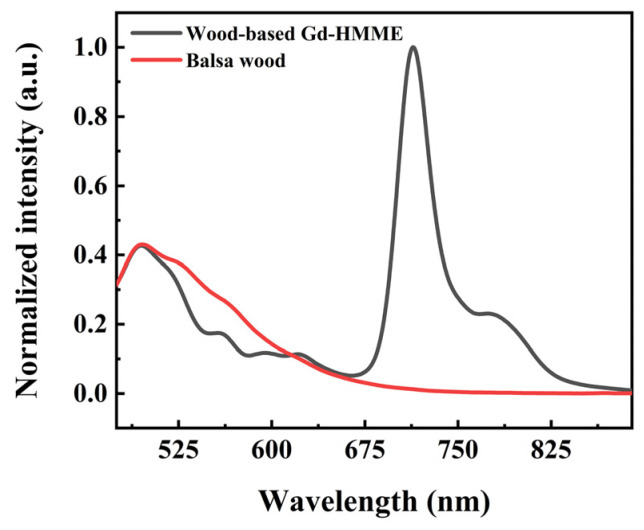
The photoluminescence spectra of balsa wood and wood-based Gd-HMME.

Wood-based Gd-HMME material was prepared via an impregnation process. To investigate the luminescence properties of the wood-based Gd-HMME material, the photoluminescence spectra of both wood-based Gd-HMME and untreated balsa wood were analyzed, as illustrated in Figure 2. The photoluminescence spectrum of untreated balsa wood exhibits broad luminescence emission from 475 nm to 662 nm. After impregnation with Gd-HMME, the photoluminescence spectrum of wood-based Gd-HMME material not only exhibited the intrinsic spectral characteristics of balsa wood with an intense emission concentrated at 475 nm, but also the spectral characteristics of Gd-HMME with two weak fluorescence emissions at 582 nm and 627 nm and strong phosphorescence emission at 711 nm and 790 nm (as shown in Figure 1b). However, within the spectral range of 514 nm to 600 nm, the luminescence intensity of the impregnated sample showed a noticeable “depression” compared to that of the unimpregnated sample. Given that the combination of balsa wood and Gd-HMME is through a physical impregnation process without chemical property changes, the noticeable depression is likely attributed to the absorption of Gd-HMME within this wavelength range. The Q-bands of Gd-HMME overlap with the fluorescence emission of balsa wood, leading to the quenching of balsa wood’s luminescence in this region. The depression spectral ranges from 514 nm to 558 nm and 558 nm to 591 nm in the wood-based Gd-HMME overlap with the two Q-bands of Gd-HMME that range from 514 nm to 558 nm and 558 nm to 591 nm. This observation further indicates that the luminescence depression from 514 nm to 591 nm of wood-based Gd-HMME is attributed to the absorption of Gd-HMME.

### 3.3. Morphological Characteristics of Balsa Wood and Wood-Based Gd-HMME

**Figure 3:**
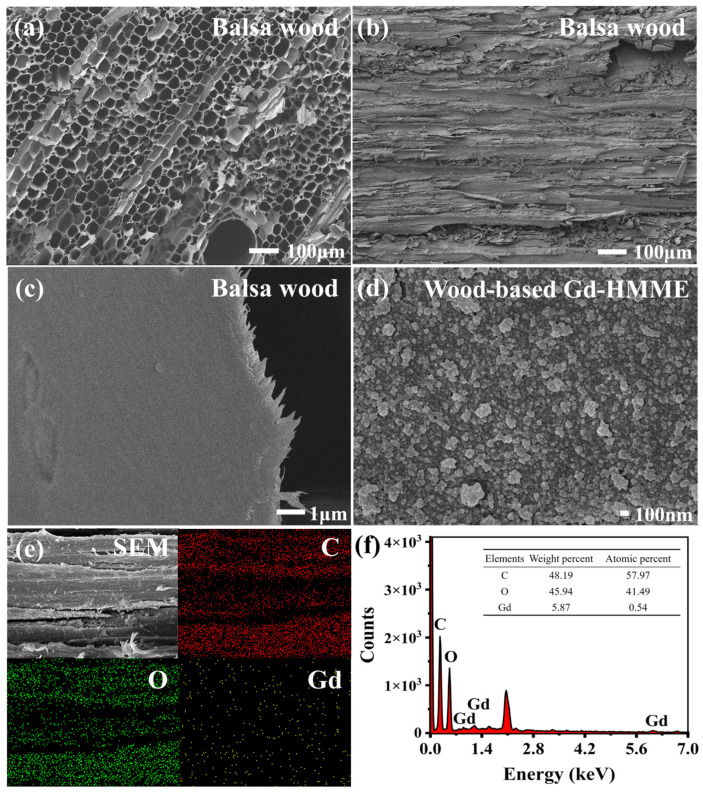
SEM and EDS images of wood-based Gd-HMME. (**a**,**c**,**d**) Cross-sectional morphology; (**b**) tangential sectional morphology; (**e**) the elemental mapping of C, O, and Gd in wood-based Gd-HMME; (**f**) the EDS spectrum of wood-based Gd-HMME. Inset: elemental content analysis.

The microstructural characteristics of both balsa wood and the wood-based Gd-HMME material were investigated, as shown in Figure 3. The cross-sectional morphology of balsa wood that was not impregnated with Gd-HMME solution is presented in Figure 3a. It can be observed that the cross-sectional microstructure exhibits a natural honeycomb-like structure, and the pores are uniformly distributed and interconnected, which provide relatively high porosity. The pore size ranges from 10 μm to 180 μm, with an average diameter of approximately 38 μm. The tangential section of balsa wood (Figure 3b) reveals a loose cellular structure with abundant, uniformly oriented pore channels, along with numerous intersecting channels. To further investigate the surface morphology of the balsa wood channels, Figure 3b is magnified, as illustrated in Figure 3c. It is evident that the channels possess a relatively smooth texture and exhibit characteristic fiber structures of wood. 

Figure 3d depicts the pore-channeled surface of balsa wood following physical impregnation with Gd-HMME. Compared to Figure 3c, Gd-HMME exhibits uniform distribution throughout the pore channels, forming a relatively stable surface adsorption layer without noticeable aggregation. Furthermore, the elemental mapping of wood-based Gd-HMME, as shown in Figure 3e, revealed a uniform distribution of carbon, oxygen, and gadolinium on the wood surface, indicating that Gd-HMME was uniformly distributed on the balsa wood. In addition, Figure 3f exhibits the EDS spectrum and elemental content analysis of wood-based Gd-HMME. It is found that there are three characteristic excitation energy peaks belonging to gadolinium; meanwhile, we discovered that the weight percentage and atomic percentage of gadolinium in the material were determined to be 5.87 and 0.54, respectively. These findings suggest that the wood-based Gd-HMME material can be effectively prepared via a physical impregnation process. 

Further analysis revealed that the average particle size of Gd-HMME is approximately 80 nm. By comparing the morphological characteristics of the balsa wood in both impregnated and unimpregnated states, it is evident that both the Gd-HMME particles and oxygen molecules are on the nanometer scale, which is significantly smaller than the pore size of balsa wood. This nanoscale compatibility not only ensures the effective adhesion of Gd-HMME molecules but also facilitates the rapid and unimpeded diffusion of oxygen molecules. Consequently, balsa wood serves as a robust structural framework for the wood-based Gd-HMME material in oxygen sensing, establishing a solid foundation for its potential applications.

### 3.4. The Relationship Between OP and Oxygen Partial Pressure Using Wood-Based Gd-HMME

**Figure 4:**
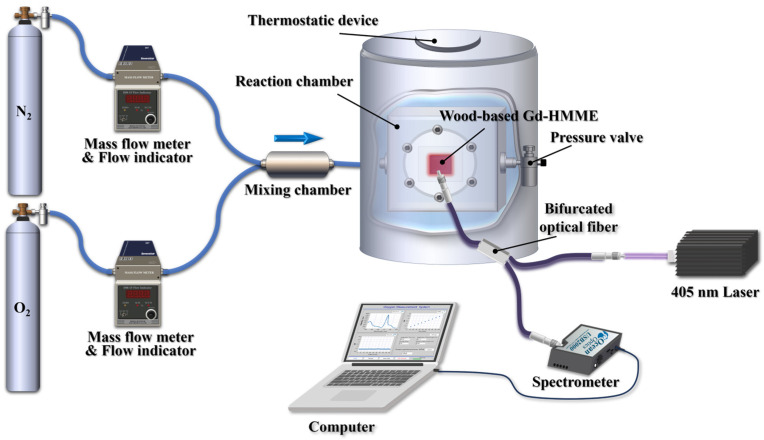
Devices for oxygen detection using wood-based Gd-HMME.

To investigate the relationship between the luminescence of wood-based Gd-HMME and oxygen, the luminescence of the material under varying oxygen levels was measured. The devices are illustrated in Figure 4. Pure nitrogen and pure oxygen were mixed to achieve varying oxygen levels using the mass flow controllers and subsequently introduced into a sealed gas chamber containing the samples for analysis. A 405 nm laser was utilized as the light source.

**Figure 5:**
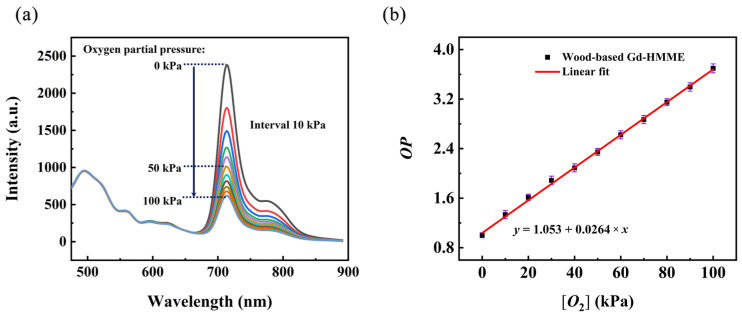
(**a**) The photoluminescence spectra of wood-based Gd-HMME at different oxygen partial pressures, (**b**) *OP* at different oxygen partial pressures.

The photoluminescence spectra of wood-based Gd-HMME material under varying oxygen partial pressures ([*O*_2_]) are presented in Figure 5a. It is evident that the spectral profile of wood-based Gd-HMME remains unchanged across different [*O*_2_]. Specifically, the luminescence intensity in the range of 475 nm to 660 nm, which corresponds to the characteristic emission of balsa wood and the fluorescence peaks of Gd-HMME, does not vary with increasing [*O*_2_]. Conversely, the luminescence intensity in the range of 660 nm to 850 nm, which is attributed to the phosphorescence emission of Gd-HMME, decreases as the [*O*_2_] increases. These observations suggest that neither the luminescence of balsa wood nor the fluorescence of Gd-HMME are influenced by the changes of [*O*_2_]; in contrast, only the phosphorescence emission of Gd-HMME changes markedly with increasing [*O*_2_]. *OP* was defined as the ratio of the phosphorescence intensity of the wood-based Gd-HMME material under anaerobic and aerobic conditions. The relationship between *OP* and [*O*_2_] is illustrated in Figure 5b. It can be observed that *OP* increases linearly with increasing [*O*_2_]. By linear fitting the experimental data, a linear relationship between *OP* and [*O*_2_] using the wood-based Gd-HMME material was established as the following equation: $OP=1.053+0.0264[O_{2}]$. This relationship conforms to the form described by the Stern–Volmer equation [40].

(1) $$OP=\frac{I_{p0}}{I_{p}}=1+K_{\mathrm{SV}}\left[O_{2}\right]$$

where *I*_p0_ is the integrated intensity of the phosphorescence peak of the wood-based material in the absence of oxygen, *I*_p_ is the integrated intensity of the phosphorescence peak in the presence of oxygen, [*O*_2_] denotes the partial pressure of oxygen, and *K*_SV_ is a proportionality constant that represents the sensitivity of oxygen detection. In this study, the *K*_SV_ value of balsa wood impregnated with 0.1 mg/mL Gd-HMME methanol solution was determined to be 0.0264.

### 3.5. Effect of Gd-HMME Concentration on the K_SV_ Value of Wood-Based Gd-HMME Material

**Figure 6:**
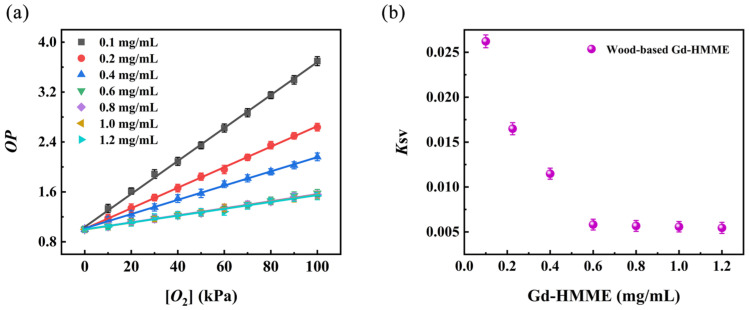
(**a**) The relationship between *OP* and oxygen partial pressure and (**b**) *K*_SV_ values at varying Gd-HMME concentrations using wood-based Gd-HMME material.

To investigate the effect of Gd-HMME concentration on the *K*_SV_ value of wood-based Gd-HMME, balsa wood was impregnated with methanol solutions of Gd-HMME at varying concentrations to prepare samples with different Gd-HMME contents. Subsequently, Stern–Volmer curves for each sample were measured, as shown in Figure 6a. As illustrated, for each sample, the *OP* value of the wood-based Gd-HMME materials exhibits a linear increase with the [*O*_2_] increases. By linearly fitting the experimental data, the *K*_SV_ values for each sample were determined, as illustrated in Figure 6b. It is evident that when the concentration of Gd-HMME is within the range of 0.1 mg/mL to 0.6 mg/mL, the *K*_SV_ value of the wood-based Gd-HMME material rapidly decreases as the Gd-HMME concentration increases; when the concentration of Gd-HMME exceeds 0.6 mg/mL, the *K*_SV_ value remains nearly constant despite further increases in the Gd-HMME concentration. In this study, the sample with the Gd-HMME concentration of 0.1 mg/mL exhibited the highest *K*_SV_ value and was therefore selected for subsequent testing.

### 3.6. Photobleaching Behavior of Wood-Based Gd-HMME Material

**Figure 7:**
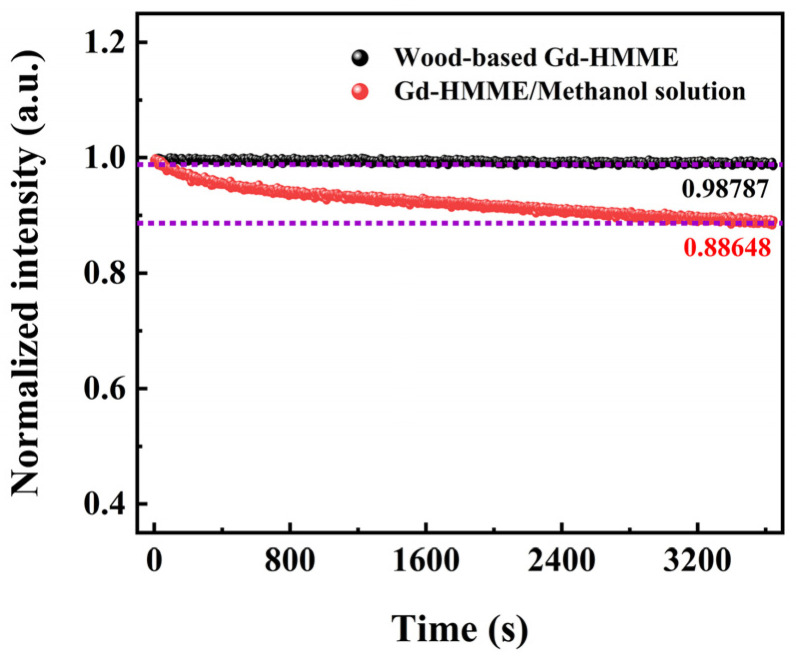
Phosphorescence intensity of wood-based Gd-HMME and Gd-HMME in methanol solution monitored at 711 nm under the irradiation of 405 nm laser for 3600 s with a power density of 1.5 mW/cm^2^.

Photobleaching behavior is a phenomenon observed in luminous materials during their application [41]. Prolonged exposure of luminous materials to light may cause a gradual reduction in luminescence intensity, leading to a decrease in the signal-to-noise ratio during the detection process. This, in turn, affects the accuracy of measurements, and this process is irreversible [42]. Therefore, evaluating the photobleaching behavior of luminous materials is crucial for their practical applications. The wood-based Gd-HMME material was continuously irradiated by a 405 nm laser for 3600 s under a high-purity nitrogen atmosphere. The laser power density was adjusted to 1.5 mW/cm^2^, and the phosphorescence intensity at 711 nm under different irradiation times is illustrated in Figure 7. The phosphorescence intensity of Gd-HMME in methanol solution was measured under the aforementioned illumination conditions for comparison. It can be observed that the phosphorescence intensity of the wood-based Gd-HMME material remains almost unchanged after continuous irradiation for 3600 s, maintaining 98% of its initial intensity. In contrast, the phosphorescence intensity of Gd-HMME in methanol solution decreased to 88% of its initial value. These results indicate that Gd-HMME exhibits markedly different photobleaching behavior in balsa wood and methanol solutions. The observed phenomenon can be attributed to the dynamic environment in methanol solution, where Gd-HMME molecules experience an increased probability of interacting with oxygen molecules, thereby accelerating the inactivation reaction. In contrast, when Gd-HMME molecules are embedded in balsa wood, the wood structure provides a rigid environment for the Gd-HMME molecules, restricting their movement and inhibiting their interaction with oxygen molecules. Therefore, the photobleaching phenomenon of Gd-HMME is more significant in solution than in wood. These findings suggest that balsa wood provides an effective photobleaching-resistant environment for Gd-HMME molecules. The wood-based Gd-HMME material can be used as a reliable candidate for continuous and stable oxygen monitoring.

### 3.7. The Response Time and Reversibility of the Wood-Based Gd-HMME Material for Oxygen Sensing

Response time and recovery time are two key indicators for evaluating the performance of the oxygen sensor. A fast response time ensures that the sensor can promptly detect any environmental changes, and a short recovery time reflects the sensor’s stability and reusability. Evaluating both response and recovery times is essential for ensuring the reliability and accuracy of sensor performance across diverse environments.

**Figure 8:**
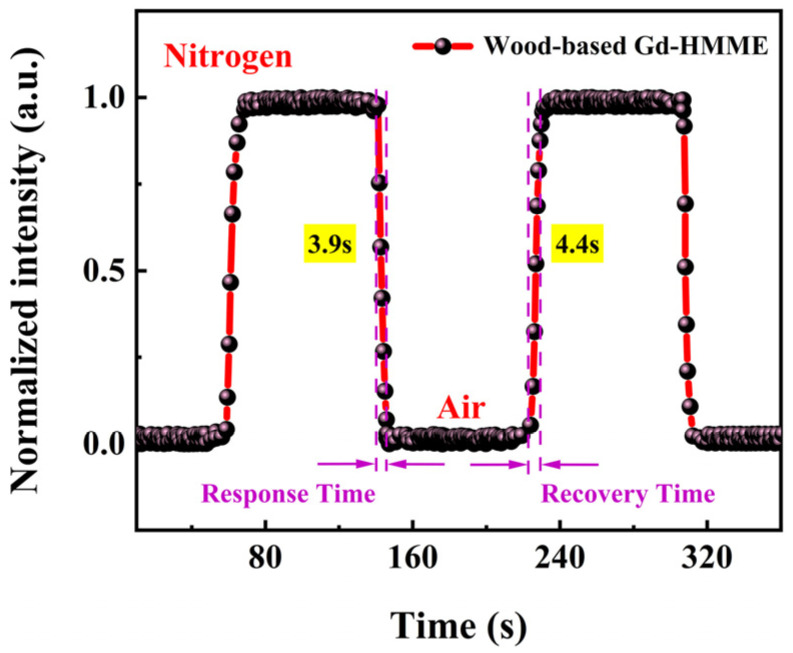
Phosphorescence intensity of wood-based Gd-HMME material monitored at 711 nm in alternating conditions between air and an anaerobic environment.

In this study, the response time and recoverability of wood-based Gd-HMME as an oxygen-sensing material were systematically evaluated. The phosphorescence intensity changes at 711 nm were continuously monitored under alternating anaerobic and air conditions, as illustrated in Figure 8. It is evident that the phosphorescence intensity in an oxygen-free environment is significantly higher compared to that in an air environment. Upon transitioning from air to an anaerobic environment, the phosphorescence intensity of wood-based Gd-HMME increased rapidly, while it decreased when the environment was switched back from anaerobic to air conditions. Over two cycles, wood-based Gd-HMME demonstrated excellent repeatability and stability. Response time is defined as the time required for phosphorescence to decrease by 95% when transitioning from an oxygen-free environment to an air environment, and the recovery time is defined as the time required for phosphorescence to return to 95% of its original intensity when transitioning from an air environment to an oxygen-free environment [43]. Wood-based Gd-HMME exhibited rapid response and recovery times and was determined to be 3.9 s and 4.4 s, respectively. These results suggest that wood-based Gd-HMME can deliver precise and timely measurement results in continuous or frequently fluctuating environments.

### 3.8. Effect of Humidity and Interference Gases on Oxygen Measurement Performance of Wood-Based Gd-HMME

In the process of oxygen detection, water vapor and common gases such as methane (CH_4_), carbon monoxide (CO), carbon dioxide (CO_2_), hydrogen (H_2_), and argon (Ar) are often present in the test samples. These additional components may lead to measurement deviations. Therefore, it is crucial to evaluate the specificity of wood-based Gd-HMME for oxygen detection.

**Figure 9:**
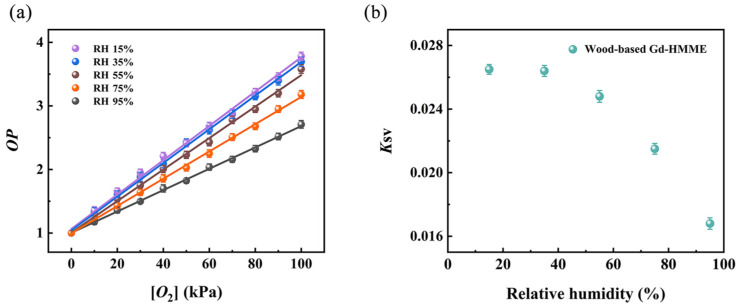
(**a**) The relationship between *OP* and oxygen partial pressure and (**b**) *K*_SV_ values at different relative humidity levels.

The *OP* values of wood-based Gd-HMME material under varying [*O*_2_] were obtained under relative humidity levels of 15%, 35%, 55%, 75%, and 95%, which are illustrated in Figure 9. The results demonstrated that *OP* increased linearly with increasing [*O*_2_] across all humidity conditions. By linearly fitting the experimental data, *K*_SV_ values were obtained for each humidity level. It was observed that *K*_SV_ values gradually decreased as humidity increased. This reduction is primarily attributed to the increased water vapor content, which hinders the interaction between Gd-HMME indicator molecules and oxygen molecules. Additionally, the increase in water vapor content also dilutes the oxygen concentration in the gas samples, further reducing the effective distribution of oxygen. Considering these two factors, humidity has a substantial impact on the oxygen sensing sensitivity of the wood-based Gd-HMME material, with higher humidity leading to lower sensitivity. To ensure the accuracy of the detection, the gas samples may need to be preprocessed using dehumidification equipment before testing.

**Figure 10:**
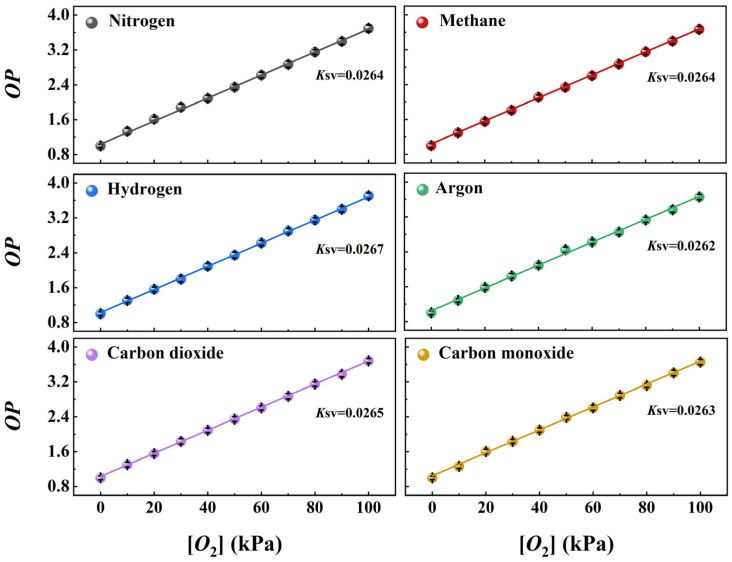
The Stern–Volmer curves of wood-based Gd-HMME material in different gas mixtures.

Furthermore, the impact of several composite gases in the air on the detection performance of wood-based Gd-HMME was evaluated. The relationship between *OP* and [*O*_2_] was investigated in different gaseous mixtures comprising various gases and oxygen, as illustrated in Figure 10. It is evident that *OP* increases linearly with the rise in [*O*_2_] across various gas mixtures. By linear fitting the experimental data, the *K*_SV_ values of wood-based Gd-HMME under different mixed gas atmospheres were determined. The results showed that the *K*_SV_ values remained nearly constant across varying gas–oxygen mixtures. These findings suggest that the gases commonly present in the atmosphere do not affect the oxygen sensing performance of wood-based Gd-HMME. Consequently, it is unnecessary to account for the influence of common atmospheric gases when measuring oxygen using wood-based Gd-HMME.

### 3.9. Long-Term Stability of the Wood-Based Gd-HMME

**Figure 11:**
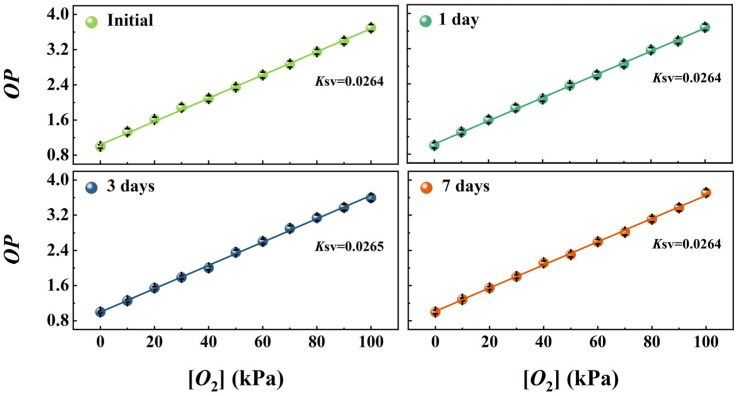
The Stern–Volmer curves of wood-based Gd-HMME as initially prepared, as well as after 1, 3 and 7 days of storage.

The long-term stability of oxygen sensing materials is critical for their practical application. Therefore, the oxygen sensing performance of the wood-based Gd-HMME material across different storage times was evaluated under a consistent relative humidity of 35%. The relationship between *OP* and [*O*_2_] was obtained as a fresh preparation, as well as after 1, 3 and 7 days of storage, shown in Figure 11. It is obvious that *OP* increased linearly with [*O*_2_] in a range of 0–100 kPa. Further, the data from the experiments were linearly fitted to obtain the values of *K*_SV_ for wood-based Gd-HMME at different storage times. From the results, it can be found that the *K*_SV_ values exhibit minimal fluctuation. The above research has demonstrated that the Gd-HMME material exhibits long-term stability in oxygen sensing and shows significant potential for practical applications.

### 3.10. Automatic Oxygen Detection System Based on Wood-Based Gd-HMME Material

**Figure 12:**
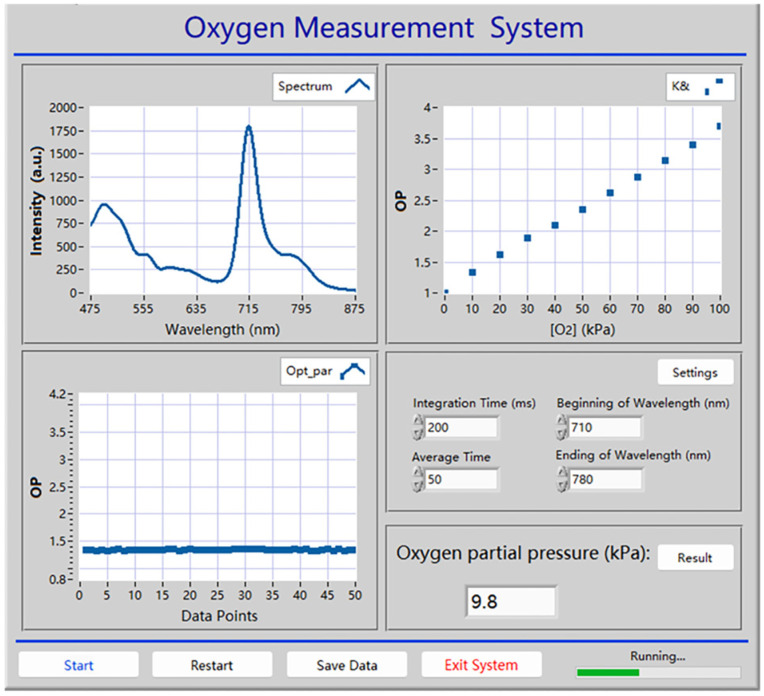
Front panel of automated oxygen detection system based on LabVIEW software.

**Figure 13:**
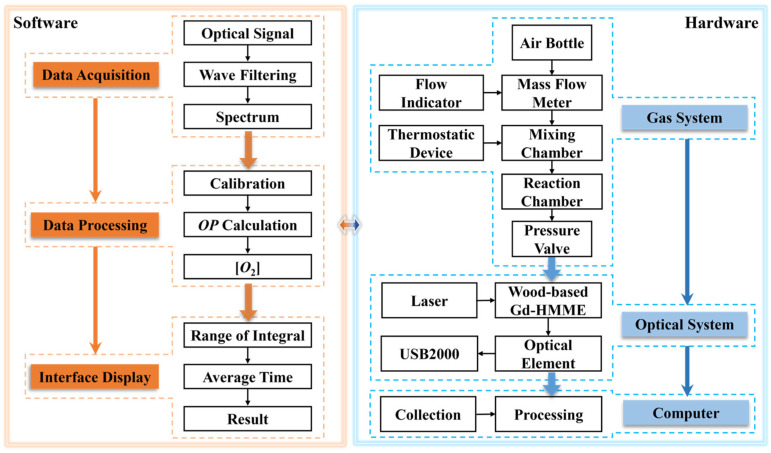
Principle block diagram of the oxygen sensing system.

To facilitate the practical application of wood-based Gd-HMME for oxygen measurement, an automatic detection system utilizing LabVIEW (version 2018) software was developed. The front panel of the detection system is exhibited in Figure 12, which comprises the spectrum acquisition system, data extraction and processing system, and parameter configuration system. The spectrum of wood-based Gd-HMME in the testing environment is captured by the spectrum acquisition system; the *OP* value is obtained by extracting the phosphorescence intensity at 711 nm; and then it is measured 50 times. The final *OP* value is calculated by averaging the experiment data. The measured oxygen concentration is derived based on the calibration curve. A principle block diagram of the oxygen detection system is shown in Figure 13. Additionally, parameters such as integration time, averaging frequency of the spectrometer (USB2000), and the display range of the spectrum can be readily adjusted.

### 3.11. Performance of the Automatic Detection System in Practical Gaseous Oxygen Detection

**Figure 14:**
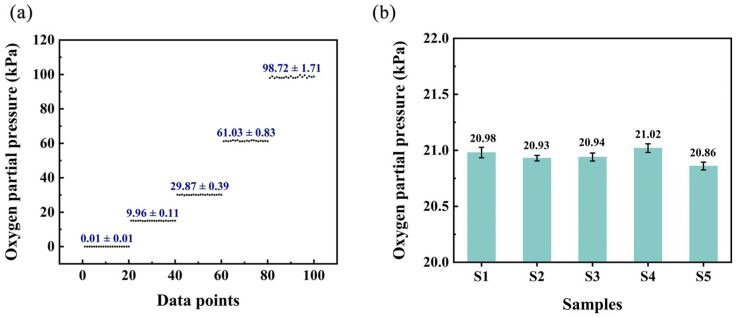
(**a**) The measured results and corresponding uncertainties of samples with different oxygen partial pressures and (**b**) the measured values on the real samples using the developed system.

The performance of the automatic oxygen detection system in practical use is further evaluated. In the practical detection process, there will be some fluctuations that may influence the detection results. To quantify these variations, the uncertainty is employed as a metric, representing the deviation of measured values from the true values. [*O*_2_] is determined through multiple measurements of the gas sample, and the uncertainty is defined as half of the range between the maximum and minimum measured values. Gas samples containing varying levels of oxygen were selected for measurement in order to assess the uncertainty of the system. Each sample was measured twenty times and the measured [*O*_2_] and corresponding uncertainty are shown in Figure 14a. It is evident that the uncertainty of the system increases as the [*O*_2_] of the test sample rises. This phenomenon can be attributed to the reduced signal-to-noise ratio of the testing equipment at higher oxygen concentrations. Typically, the oxygen concentration at which the change in the *OP* value equals the standard deviation is defined as the detection limit of the system. In this study, the detection limit was determined to be 0.01 kPa. 

Additionally, the system was applied to real samples to evaluate its accuracy for oxygen sensing. Five test samples were collected from the atmosphere at different timepoints throughout the day, specifically at 7:00, 11:00, 15:00, 19:00, and 23:00. The automatic oxygen sensing system was applied to test the oxygen levels in the test samples and the results are shown in Figure 14b. The measurement values exhibit a high degree of consistency with the theoretical value (20.95%). These findings not only demonstrate the system’s reliability but also demonstrate the potential of wood-based Gd-HMME as an effective oxygen sensing material. Ultimately, the oxygen sensing performance of the wood-based Gd-HMME was compared with that of other reported oxygen sensing materials; these results are summarized in Table 1.

## 4. Conclusions

In this study, balsa wood was chosen as the supporting matrix to prepare a wood-based Gd-HMME oxygen sensing material through physical impregnation with Gd-HMME. The oxygen-sensing performance of the materials was systematically evaluated. The results of SEM and EDS indicated that balsa wood exhibits a large and abundant porous structure, with Gd-HMME adhering to the pore surfaces uniformly. *OP* is defined as the ratio of phosphorescence intensity of the wood-based Gd-HMME material under anaerobic and aerobic conditions. A linear relationship between *OP* and oxygen partial pressure ([*O*_2_]) within the range of 0–100 kPa was obtained, which is consistent with the Stern–Volmer curve. The *K*_SV_ value was revealed to be decreased initially and subsequently unchanged as the Gd-HMME concentration increased. The optimal Gd-HMME concentration was determined to be 1.0 mg/mL, and the calibration equation between *OP* and [*O*_2_] was established as $OP=1.053+0.0264[O_{2}]$.

The wood-based Gd-HMME material was continuously irradiated to assess its photobleaching behavior. The phosphorescence intensity was found to be almost unchanged after 3600 s of continuous irradiation by a 405 nm laser, which verified the excellent photobleaching resistance of the material. The wood-based Gd-HMME exhibited a rapid response time of 3.9 s and efficient recovery, enabling reliable and repeated measurements in complex environments. Additionally, the impact of the relative humidity and interfering gases on oxygen measurement using the wood-based Gd-HMME was evaluated. The results indicated that the *K*_SV_ value of the material decreased significantly as the relative humidity increased from 15% to 95%, demonstrating that air humidity substantially affects the measurement outcomes. In contrast, the presence of interfering gases did not influence the specificity of the oxygen measurements. Additionally, the wood-based Gd-HMME material was stored in the dark to evaluate the long-term stability of the material. The results indicated that the performance of the wood-based Gd-HMME material remained stable for 7 days. Furthermore, an automatic oxygen detection system was developed using LabVIEW (version 2018) software, and it was employed to assess the fluctuations encountered during practical applications. These results indicated that the fluctuation intensified as the [*O*_2_] increased. This phenomenon is attributed to the reduced signal-to-noise ratio of the spectrometer at higher oxygen concentrations; the detection limit was determined to be 0.01 kPa. Finally, the automatic oxygen sensing system demonstrated high accuracy in tests with real samples. The wood-based Gd-HMME material developed in this study supports oxygen measurement in a broad range of 0–100 kPa and is well-suited for detecting complex and dynamic gaseous oxygen environments. Future research will focus on enhancing the hydrophobic properties of wood-based Gd-HMME materials, thereby extending their application to the detection of dissolved oxygen concentrations in liquids.

## Figures and Tables

**Table 1:** Comparison of the performance of different oxygen sensors.

Matrix and Dye	Detection Range (kPa)	Limit of Detection	*K*_SV_ (kPa^−1^)	Response Time	Reference
PS and PtTPP	5–20	-	<0.85	<60 s	[44]
Fiber and PtOEP	0–60	-	-	<164 s	[45]
tButPS and PtTPTBPF_4_	0–55	-	0.477	<10 s	[46]
EC and PtOEP	0–70	-	0.298	3 s	[47]
PMMA and TA2P	0–1.61	0.0979 Pa	10.22	-	[48]
Methanol and Gd-HMME	0–8	~0.03 kPa	0.124	-	[32]
Filter paper and Gd-HMME	10–100	-	~0.016	~0.4 s	[28]
Balsa wood and Gd-HMME	0–100	0.01 kPa	<0.0264	~3.9 s	This work

## Data Availability

The data presented in this study are available on request from the corresponding author.

## Abbreviations

The following abbreviations are used in this manuscript:

Gd-HMME	Gadolinium-labeled hematoporphyrin monomethyl ether
HMME	Hematoporphyrin monomethyl ether
SEM	Scanning electron microscope
EDS	Energy dispersive spectrometer
*OP*	Optical parameter
[*O*_2_]	Oxygen partial pressure
*K*_SV_	Stern–Volmer constant
RH	Relative humidity
